# Seroepidemiology of Hepatitis B and C Virus Infections: A Five-Year Retrospective Study among Blood Donors in Saboba District in the Northern Region of Ghana

**DOI:** 10.1155/2021/5599705

**Published:** 2021-05-11

**Authors:** Abel Makija Nlankpe, Precious Kwablah Kwadzokpui, David Adedia, Jacob Nabei Nignan, Patrick Kwasi Owiafe

**Affiliations:** ^1^Department of Medical Laboratory Sciences, School of Allied Health Sciences, University of Health and Allied Sciences, Ho, Ghana; ^2^Reinbee Medical Laboratory and Wellness Center, Ho, Ghana; ^3^Department of Basic Sciences, School of Basic and Biomedical Sciences, University of Health and Allied Sciences, Ho, Ghana; ^4^Assemblies of God Hospital, Saboba, Ghana

## Abstract

**Methods:**

A five-year hospital-based retrospective study was carried out among 8605 blood donors comprising 8517 males and 88 females using data on blood donors from Saboba Assemblies of God Hospital located in the Saboba District in the Northern Region of Ghana from 2013 to 2017. Blood bank records on HBV and HCV potential blood donors who visited the hospital to donate blood were retrieved. Donor demographic details, i.e., age and gender, were also recovered. Donors who were registered to the hospital but were not residents of the Northern Region were excluded from the study. Donors with incomplete records were also excluded from the study. The data was managed using Microsoft Excel spreadsheet 2016 and analysed using GraphPad Prism statistical software.

**Results:**

The overall prevalence of asymptomatic viral hepatitis B and C infections in the general adult population was 9.59% (95% CI: 9.00-10.20) and 12.71% (95% CI: 12.00-13.40), respectively, with an HBV/HCV coinfection rate of 2.23% (95% CI: 1.90-2.60). The number of donors generally declined with advancement in years from 2038 (23.68%) since 2013 to as low as 1169 (13.59%) in 2016, except for 2017 where a sharp increase of 1926 (22.38%) was observed. The first and second highest proportions of donors fell within the age categories of 20-29 (51.53% (4434)) and 30-39 (32.90% (2831)) respectively. The seroprevalence rate of HBV, HCV, and HBV/HCV coinfection rates were generally higher among the female group than those observed among the male category. The year-to-year variation in HBV, HCV, and HBV/HCV infections was statistically significant. The highest year-to-year HBV seropositivity rate was 11.48% in the year 2013, while that for HCV and HBV/HCV coinfection was 16.24% and 5.85%, respectively, both documented in the year 2014. HBV and HBV/HCV coinfection rates were highest among donors aged <20 years old, while HCV seroprevalence was highest among donors aged 50-59 years old. Significantly higher odds of HBV/HCV coinfection (OR = 5.2; 95% CI:3.3-8.1) was observed in the 2014 compared to the year 2013. Donors aged <20years were at higher risks of HBV and HBV/HCV coinfection rates compared to the other age groups.

**Conclusion:**

The seroprevalence of HBV and HCV among donors in the Saboba District of the Northern Region of Ghana is endemic. The HBV/HCV coinfection rate also raises serious concern owing to its high prevalence rate among the younger age. Intensive public health education coupled with mobile screening and mass vaccination of seronegative individuals is advised so as to help curb further spread of the infection and in effect help safeguard the health status of potential donors in the district.

## 1. Introduction

Hepatitis, the viral type to be specific, refers to the inflammation of the hepatocytes as a result of viral infection. Types A, B, and C of the viral hepatitis are the three common types among the five types of the hepatitis viruses vis-à-vis hepatitis A, B, C, D, and E. Hepatitis B virus (HBV) infection is a major public health problem worldwide infecting about 30% of the world's population [1, 2]. This infection has been ranked the 15th cause of death in all cases of global mortality [3]. HBV infection is associated with an increased risk of cirrhosis, hepatic decompensation, and hepatocellular carcinoma (HCC) in 15%-40% of infected subjects [4, 5]. The World Health Organization (WHO) has estimated that 2 billion people worldwide have been infected with HBV and that about 350-400 million of these people are chronically infected, approximately 65 million of which are projected to die from liver disease due to the infection [2, 4, 6]. The disease has been documented to cause between 600,000 and 800,000 deaths every year [3, 7–10]. In Sub-Saharan Africa, about 8% of the population is chronic carriers of HBV with the highest rate of 2.8% in the West African subregion, including Ghana, where an estimated national prevalence of 12.3% in a systemic review has been documented [11]. Infection with the hepatitis C virus (HCV) and the associated pathological outcome are analogous to those of HBV infection. When not detected early for treatment, HCV infection can lead to complications such as liver cirrhosis and hepatocellular carcinoma (HCC), which have been tagged as the principal cause for liver transplant in the United States [12]. Of global concern is the fact that Shepard and colleagues in their study implicated HCV infection as a major potential cause of substantial morbidity and mortality in the future [13]. According to the WHO, 71 million of the world's population is chronically infected with HCV, resulting in approximately 399,000 fatalities annually [14], 5.3% of which has been reported in Sub-Saharan Africa [15, 16]. A population-based epidemiological study conducted recently in Iran, a country categorised as low-endemic country, revealed an increasing burden of the disease among the Iranian people [17]. Further studies revealed that this saliently elevating trend in HCV infection was largely contributed by an increase in the activities of injection drug users [17, 18]. Here in Africa, Madhava and colleagues indicated that Central Africa has the highest estimated HCV prevalence of 6% while 2.4% and 1.6% were reported in West Africa and Southern and Eastern Africa, respectively [15]. The prevalence of HCV in the general population in Africa ranges between 0.1% and 17.5%, the highest prevalence in Egypt (17.5%), 13.8% in Cameroon, and 11.3% in Burundi with lower prevalence rates recorded in Zambia, Kenya, Malawi, and South Africa among others [19]. Regional prevalence of chronic HCV infection was determined for the Ashanti region as 1.5% to 1.9% and Greater Accra region as 6.4% to 8.6% [20]. In Ghana, there is no published literature on the prevalence of HBV and HCV coinfections in the Northern region, particularly the Saboba District. This study therefore sought to determine the prevalence and yearly trend of hepatitis B and C coinfections among blood donors in Saboba District, Ghana.

## 2. Materials and Methods

### 2.1. Study Design/Eligibility Criteria

A five-year hospital-based retrospective study was carried out among 8605 blood donors comprising 8517 males and 88 females using data on blood donors from Saboba Assemblies of God Hospital located in the Saboba District in the Northern Region of Ghana from 2013 to 2017. The hospital is a 124-bed capacity facility made up of the adult male ward (30 beds), children's ward (33 beds), neonatal intensive care unit (19 beds), maternity ward (20 beds), female ward (22 beds) and a theatre, laboratory, radiography unit, the outpatient department (OPD), consulting rooms, the dispensary, and an administration block. Blood bank records on HBV and HCV potential blood donors who visited the hospital to donate blood were retrieved. Donor's demographic details, i.e., age and gender, were also retrieved. Test kits/strips employed for the serological diagnosis of the two viral infections were those produced from the following companies: DiaSpot (cassette type) in 2013 and 2014 (99% sensitivity and 97% specificity), ACON (strips) in 2015 (93% sensitivity and 100% specificity), ABON (strips) in 2016 (99% sensitivity and 99% specificity), and OneStep HIGHTOP (strips) from 2017 till date (99.3% sensitivity and 99.2% specificity). Donors who were registered with the hospital but were not residents of the Northern Region were excluded from the study. Donors with incomplete records were also excluded from the study.

### 2.2. Data Collection and Analysis

Due to the large volume of data, a fit-for-purpose Microsoft Excel form was employed to capture the data into Microsoft Excel spread sheet version 2016. The data after capturing using the Excel software was checked for completeness and then transferred into IBM SPSS statistical software for statistical analysis. Binary logistics regression analysis was employed to determine the likelihoods of infection. The Clopper-Pearson test statistic was used to determine the 95% confidence intervals of the observations made. Results are presented in the form of tables and figures. The Chi-square (*χ*^2^) test was employed to determine the statistical significance among variables under review. *p* value ≤ 0.05 were considered statistically significant.

## 3. Results

Of the overall 8605 prospective blood donors recruited in this study, 9.59% (95% CI; 9.00-10.20), 12.71% (95% CI; 12.00-13.40), and 2.23% (95% CI; 1.90-2.60) reported positive for HBV, HCV, and HBV/HCV coinfections, respectively. Number of donors generally declined with advancement in years from 2038 (23.68%) since 2013 to as low as 1169 (13.59%) in 2016 except for 2017 where a sharp increase of 1926 (22.38%) was observed. Seropositive HBV prevalence has also been on a downward trajectory since 2013 with 11.48% rate to 7.61% in 2016 but saw a slight upsurge of 8.20% in the year 2017 compared to 2016. Seroprevalence rates of HCV infection on the other hand has been somewhat wavy over the five-year period with the highest prevalence rate of 16.24% occurring in 2014 and the lowest, 7.78%, in 2016. The second and third highest HCV prevalence rates over the five-year period were observed in the years 2013 and 2017, respectively. HBV/HCV was 1.18% in 2013 until a sharp increase of 5.85% was observed in 2014 followed by a constant decline to as low as 0.99% in 2017. Compared to 2013, the likelihood of HBV and HCV infections generally decreased as the years went by except for 2014 where a 10% increase in HCV infection was observed. However, the likelihood of HBV/HCV coinfection rates increased to about five times (OR = 5.2; 95% CI: 3.3-8.1) in 2014 compared to the year 2013. A 20% increase in the likelihood of HBV/HCV coinfection rate was also observed in the year 2015. The year-on-year prevalence rates of HBV, HCV, and HBV/HCV coinfections were significantly different over the period (*p* < 0.05) (Table 1).

The age-based stratification of donors revealed the first and second highest proportions of donors falling in the age categories of 20-29 (51.53% (4434)) and 30-39 (32.90% (2831)). Blood donation is less patronized by individuals aged <20 (5.66%) and ≥50 (1.48%) years old. HBV seropositive prevalence generally decreases significantly (*p* < 0.0001) with advancement in age, with the highest prevalence rate documented among donors aged <20 years old (16.84%) followed by 10.37%, 7.81%, 7.72%, and 5.17% among donors aged 20-29, 30-39, 40-49, and 50-59 years old, respectively. No donor 60 years and above was diagnosed with HBV infection. The odds of HBV infection in this study was approximately four times (OR = 3.7; 95% CI: 1.6-8.7; *p* < 0.05) higher among donors aged <20 years and approximately two times higher among donors aged 20-29 years (OR = 2.1; 95% CI: 0.9-4.9; *p* > 0.05), 30-39 years (OR = 1.6; 95% CI: 0.7-3.6; *p* > 0.05), and 40-49 years (OR = 1.5; 95% CI: 0.6-3.6; *p* > 0.05) compared to donors aged 50-59 years old. HCV infection was surprisingly highest among the extreme age categories vis-a-vis the highest 18.10% among participants aged 50-59 years followed by 17.86%, 17.79%, and 16.67% among donors aged <20, 40-49, and **≥**60 years old, respectively. The HCV seroprevalence is significantly different among the various age groups studied. Less likelihood of HCV infection was observed across all age groups when compared to donors aged 50-59 years old. Donors aged <20 years recorded the highest HBV/HCV coinfection rate of 4.93%, while those aged 50-59, 40-49, and 20-29 recorded comparable prevalence rates of 2.59%, 2.21% and 2.10%, respectively. The HBV/HCV coinfection rate in this study was statistically significant across the age categories. The likelihood of HBV/HCV coinfection was twice (OR = 2.0; 95% CI: 0.6-6.6; *p* > 0.05) and 30% higher (OR = 1.3; 95% CI: 0.1-26.6; *p* > 0.05) among donors aged <20 years and **≥**60 years, respectively, compared to donors aged 50-59 years old (Table 2).

**Figure 1:**
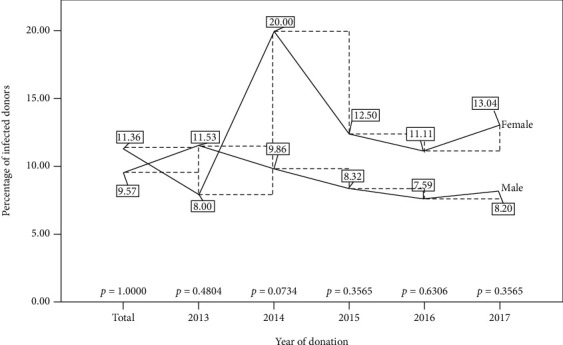
Gender distribution for the trend of HBV infection for the five-year period.

Overall, the HBV prevalence rate of 11.36% among females was documented, higher than the 9.57% prevalence in their male counterparts. This study generally saw a consistently higher HBV prevalence rate among females than observed among the males except for the year 2013 where HBV prevalence was highest among males (11.53%) than observed among females (8.00%). The year 2014 saw in proportion the highest HBV prevalence rate of 20.00% among females higher than the 9.86 percent documented among their male counterparts in the same year. The years 2015 and 2016 saw a general steady decline in the seroprevalence rates of HBV followed by a slight increase in the year 2017. Of note, however, is the fact that the rate of infection over the period has remained comparable between the two gender categories (Figure 1).

**Figure 2:**
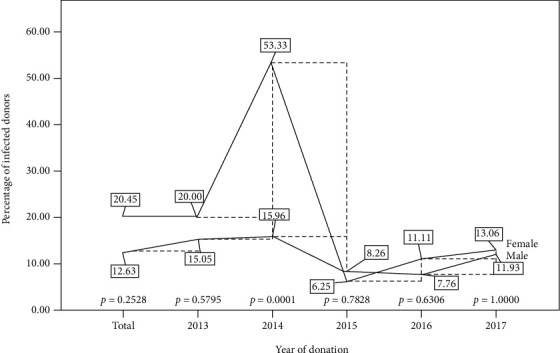
Gender distribution for the trend of HCV infection for the five-year period.

Overall, the HCV prevalence rate of 20.45% among females was documented, higher than the 12.63% prevalence in their male counterparts. The highest significant prevalence of infection (53.33%) was observed among females in the year 2014, the same year where the infection rate among their male counterparts stood at 15.95%. A sharp decline in the prevalence rate was observed among both genders in the year 2015 and then remained slightly on the upward course until 2017 (Figure 2).

**Figure 3:**
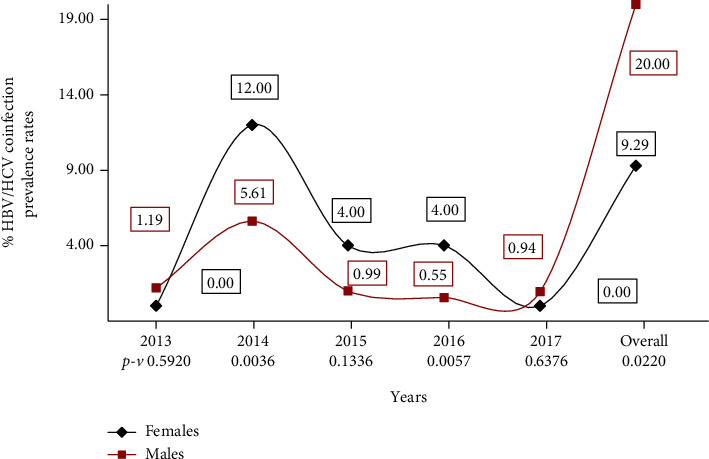
Gender distribution for the trend of HBV/HCV coinfection for the five-year period.

This study recorded an overall HBV/HCV coinfection rate of 20.00% and 9.29% among males and females, respectively, who reported to the blood bank for the purposes of donating blood. In the year 2013, the HBV/HCV coinfection rate was as low as 1.19% and 0.00% among males and females, respectively. Immediately after 2013, a significantly sharp upsurge in the HBV/HCV coinfection rate was observed among the female group (12.00%), significantly higher than the male category (5.61%). A steady but way-like decline in the HBV/HCV coinfection rate after 2015 was observed till as low as 2017 where females recorded once again a 0.00% prevalence against 0.94% among their male counterparts (Figure 3).

## 4. Discussion

The life-saving medical interventions of blood transfusion therapy cannot be underestimated. However, recipients of transfusion risk become infected with bloodborne pathogens. Despite being a life-saving intervention, the challenges posed by the process continue to be a public health threat in most countries, particularly those in developing settings. This study focused on determining the seroepidemiology of hepatitis B and C virus infections among blood donors in Saboba District in the Northern Region of Ghana.

Of the overall 8605 prospective blood donors recruited in this study, 9.59% (825/8605), 12.71% (1094/8605), and 2.23% (192/8605) reported positive for HBV, HCV, and HBV/HCV coinfections, respectively. Evidently, the prevalence of HBV and HCV is higher than the estimated global 7% (350 million) HBV and 3% HCV prevalence rates [21]. Recent studies showed higher prevalence rates of HBV than the HCV [22–24] contrary to the findings in this work. In Apollo Hospitals, Dhaka, low seroprevalence rate of HBV and HCV among all blood donors between 2007 and 2011 [25] was documented similar to that of Southeastern Nigeria [26]. Among comparable population in South of Iran, HBV and HCV prevalence rates of 0.15% and 0.004% were reported [27]. Compared to findings from this study, a similar study conducted among blood donors in a rural Ghanaian community about a decade ago revealed a higher HBV and a lower HCV, and HBV/HCV coinfection prevalence rate of 10.53%, 5.63%, and 2.09%, respectively [22]. The discrepancies in the results juxtaposed to findings from this study could be due to among many factors the geographical area, level of awareness among study subjects on issues of the infections and their mode of transmission as well as the detection methods employed in testing for the virus. Owing to the established age-associated decline in the prevalence of both HBV and HCV infections [28], it was expected that the seroprevalence rates of these viral agents would exhibit a downward trajectory with advancement in years. It was therefore not surprising to see a smooth decline in the seroprevalence rates of HBV from 11.48% to 8.20% and HCV from 15.11% to 11.94% despite the fact that the recorded prevalence rates of these infections remain very high [2, 22]. Indeed, this observation was further justified by the general decline in the odds of both HBV and HCV yearly infection rates as well as with advancement in age. It appears however that efforts to aid combat the menace of these viral infections in recent times have been shifted more towards that of HBV infections than HCV. In fact, most public health educations and screening programs prioritize HBV infection than HCV. These observations by authors over the years seem to justify the reason for the elevated HCV infections than HBV observed in this study. It is important to note that HCV is up to 4 times more infectious than the human immunodeficiency virus (HIV), requiring less exposure than HIV to cause infection [19] and therefore need to be given the needed attention as much as given other viral agents vis-à-vis HBV and HIV. In this study, the HBV/HCV coinfection rate was 1.18% in 2013 until a sharp increase of 5.85% was observed in 2014 believed to be contributed by the spike in HCV infection in same year, 2014. The year-on-year prevalence rates of HBV, HCV, and HBV/HCV coinfections were significantly different over the period (**p** < 0.05).

The age-based stratification of donors revealed the first and second highest proportions of donors falling in the age categories of 20-29 (51.53% (4434)) and 30-39 (32.90% (2831)). Blood donation was less patronized by individuals aged <20 (5.66%) and ≥50 (1.49%) years old. While HBV seroprevalence generally decreased significantly (**p** < 0.0001) from as high as 16.84% among donors aged <20 years to 5.17% and 0.00% among donors aged 50-59 and ≥60 years, respectively, with advancement in age, HCV seroprevalence demonstrated a rather wavy-like trend of infection mostly on the high with donors aged 50-59 years recording the highest prevalence rate of 18.10% followed closely by 17.86% (<20 years), 17.79%, and 16.67%. This finding served as a confirmatory information buttressing the argument that little attention is given to HCV infection in Ghana. It is important to note that home-based delivery is a common practice in Northern Ghana mostly as a result of (1) difficulties in assessing the nearest health facilities for antenatal and postnatal care in the district and (2) preference for local birth treatment to that of health facilities by gravid women. This therefore exposes the newly born to these viral agents after delivery as well as failure on the part of parents to subject the newly born to laboratory tests to ensure early diagnosis of these infections for appropriate treatment to commence upon a positive test. Relevant health officials and stakeholders need therefore to go back to the drawing board and ensure that discussion on infection prevention is rekindled, paying key attention to that of HCV to prevent a future epidemic of the viral agent among the life-saving donor population which is already a struggle by blood bank donor organizers in Ghana to come by. Another worrying development is an HBV/HCV coinfection rate of 4.93% among donors aged <20 years in this study. The authors consider this situation worrying owing to the fact that the occurrence of coinfection of HBV with other viruses such as HIV and HCV most often than not triggers an accelerated progression of liver disease with adverse clinical outcomes [7]. This does not only threaten to worsen the general health status of the population in the catchment area and the country at large but also may cause a reduction in the eligible donor population of the region.

This study reveals a higher burden of viral hepatitis infections among females than observed among their male counterparts comparable to that of Nkrumah and colleagues [22]. In contrast, Alomatu et al. and Walana et al. recorded in their works a higher prevalence of viral hepatitis B and C among males compared to their female counterparts [23, 24]. It is not quite clear from the present study what exactly could account for the female preponderance to viral infectivity but Lokpo and colleagues [11] indicated that the high infectivity of the virus could include early onset of sexual activity, wider surface area of the vagina, longer semen-vaginal contact, lower level of education, iatrogenic infection in health facilities where women are more often admitted than men especially during pregnancy and at delivery, occupation, and a lower standard of living. The high female prevalence rate in this study could also be a consequence of the small number of female blood donors, i.e., 88 out of the 8605 total donors recruited in this study as a single infection in the female population could be significant compared to the male population. This study recorded an overall HBV/HCV coinfection rate of 20.00% among males higher than 9.29% recorded among females. However, except for 2013 where the HBV/HCV coinfection rate was as low as 1.19% and 0.00% among males and females, respectively, a significantly sharp upsurge in the HBV/HCV coinfection rate of 12.00% for the year 2014 and 4.00% for the years 2015 and 2016 was observed among the female group higher than the 5.61%, 0.99%, and 0.55% prevalence rates among the male category for the years 2014, 2015, and 2016, respectively.

## 5. Conclusion

The seroprevalence of HBV and HCV among donors in the Saboba District of the Northern Region of Ghana is endemic. The HBV/HCV coinfection rate also raises serious concerns owing to its high prevalence rate among the younger age.

### 5.1. Limitations

Owing to the fact that data used in this study was obtained from the local repository (book storage) of the hospital, undetected entry errors might affect the final outcome of the study.

### 5.2. Recommendations

Intensive public health education coupled with mobile screening and mass vaccination of seronegative individuals is advised to help curb further spread of the infection and in effect help safeguard the health status of potential donors in the district. Pregnant women within the district and the country at large must be educated on the importance of delivering in a health facility.

## Figures and Tables

**Table 1:** Year-on-year trend of HBV and HCV infections among blood donors in Saboba District.

Parameter	Donors	*n* (HBV (95% CI))	OR (95% CI)	*n* (HCV (95% CI))	OR (95% CI)	*n* (coinfection (95% CI))	OR (95% CI)
Total	8605 (100.00)	825 (9.59 (9.00-10.20))		1094 (12.71 (12.00-13.40))		192 (2.23 (1.90-2.60))	
2013	2038 (23.68)	234 (11.48 (10.10-12.90))	1	307 (15.11 (13.50-16.70))	1	24 (1.18 (0.80-1.0))	1
2014	1983 (23.04)	197 (9.93 (8.70-11.30))	0.9 (0.7-1.0)	322 (16.24 (14.60-17.90))	1.1 (0.9-1.3)	116 (5.85 (4.90-7.00))	5.2 (3.3-8.1)^∗^
2015	1489 (17.30)	147 (9.87 (8.40-11.50))	0.8 (0.7-1.1)	145 (9.74 (8.30-11.40))	0.6 (0.5-0.8)^∗^	21 (1.41(0.90-2.10))	1.2 (0.7-2.2)
2016	1169 (13.59)	89 (7.61(6.20-9.30))	0.6 (0.5-0.8)^∗^	91 (7.78(6.30-9.50))	0.5 (0.4-0.6)^∗^	12 (1.03 (0.50-1.80))	0.9 (0.4-1.7)
2017	1926 (22.38)	158 (8.20 (7.00-9.50))	0.7 (0.6-0.9)^∗^	229 (11.94 (10.50-13.40))	0.8 (0.6-0.9)^∗^	19 (0.99 (0.60-1.50))	0.8 (0.5-1.5)
*p* value		<0.0001		<0.0001		0.0010	

Data are presented as frequency with corresponding percentages in parenthesis for donors. HBV, HCV, and HBV/HCV coinfections are presented as frequency and percentages in parentheses with the 95% confidence intervals attached in parenthesis. HBV: hepatitis B virus; HCV: hepatitis C virus; OR: odds ratio; CI: confidence interval. ^∗^Significant odds ratio. *p* is significant at < 0.05.

**Table 2:** Year-on-year trend of HBV and HCV Infections among blood donors of the various age groups in Saboba District.

Parameter	Donors (*n* = 8605)	*n* (HBV (95% CI))	OR (95% CI)	*n* (HCV (95% CI))	OR (95% CI)	*n* (coinfection (95% CI))	OR (95% CI)
<20	487 (5.66)	82 (16.84 (13.60-20.50))	3.7 (1.6-8.7)^∗^	87 (17.86 (14.60-21.60))	1.0 (0.6-1.7)	24 (4.93 (3.20-7.20))	2.0 (0.6-6.6)
20-29	4434 (51.53)	460 (10.37 (9.50-11.30))	2.1 (0.9-4.9)	516 (11.64 (10.70-12.60))	0.6 (0.4-1.0)	93 (2.10 (1.70-2.60))	0.8 (0.3-2.6)
30-39	2831 (32.90)	221 (7.81 (6.80-8.90))	1.6 (0.7-3.6)	339 (11.97 (10.80-13.20))	0.6 (0.4-1.0)	56 (1.98 (1.50-2.60))	0.8 (0.2-2.5)
40-49	725 (8.43)	56 (7.72 (5.90-9.90))	1.5 (0.6-3.6)	129 (17.79 (15.10-20.80))	0.9 (0.6-1.6)	16 (2.21 (1.30-3.60))	0.9 (0.2-3.0)
50-59	115 (1.34)	6 (5.22 (1.90-11.00))	1	21 (18.26 (11.70-26.50))	1	3 (2.61 (0.50-7.40))	1
≥60	13 (0.15)	0 (0.00)-	0.7 (0.0-12.8)	2 (15.38 (1.90-24.70))	0.9 (0.2-4.4)	0 (0.00)-	1.3 (0.1-26.6)
*p* value		0.0032		<0.0001		<0.0001	

Data are presented as frequency with corresponding percentages in parenthesis for donors. HBV, HCV, and HBV/HCV coinfections are presented as frequency and percentages in parentheses with the 95% confidence intervals attached in parenthesis. HBV: hepatitis B surface antigen; HCV: hepatitis C virus; OR: odds ratio; CI: confidence interval. ^∗^Significant odds ratio. *p* is significant at < 0.05.

## Data Availability

Data is available on reasonable request.
